# Effect of chemotherapy and radiotherapy on cognitive impairment in colorectal cancer: evidence from Korean National Health Insurance Database Cohort

**DOI:** 10.4178/epih.e2021093

**Published:** 2021-11-02

**Authors:** Kwanghyun Kim, Chang Woo Kim, Aesun Shin, Hyunseok Kang, Sun Jae Jung

**Affiliations:** 1 Department of Preventive Medicine, Yonsei University College of Medicine, Seoul, Korea; 2 Department of Public Health, Yonsei University, Seoul, Korea; 3 Department of Surgery, Ajou University School of Medicine, Suwon, Korea; 4 Department of Preventive Medicine, Seoul National University College of Medicine, Seoul, Korea; 5 Department of Medicine, University of California, San Francisco, CA, USA

**Keywords:** Chemotherapy, Radiotherapy, Cognitive impairment, Colorectal neoplasms

## Abstract

**OBJECTIVES:**

We investigated the risk of chemotherapy-related and radiotherapy-related cognitive impairment in colorectal cancer patients.

**METHODS:**

Medical use data of colorectal cancer patients were obtained from the Korean National Health Insurance Database from 2004 to 2018. We randomly selected 40% of all colorectal cancer patients (n=148,848). Cognitive impairment was defined as having 1 or more International Classification of Diseases, 10th revision diagnostic codes for dementia or mild cognitive impairment. Patients aged 18 years or younger, patients diagnosed with cognitive impairment before colorectal cancer diagnosis (n=8,225), and patients who did not receive primary resection (n=45,320) were excluded. The effects of individual chemotherapy regimens on cognitive impairment were estimated. We additionally estimated the effect of radiotherapy in rectal cancer patients. Time-dependent competing risk Cox regression was conducted to estimate the overall and age-specific hazard ratios (HR) separately for colon and rectal cancer. Landmark analyses with different lag times were conducted as sensitivity analyses.

**RESULTS:**

Chemotherapy did not increase the risk of cognitive impairment in colorectal cancer patients (colon cancer: HR, 0.92; 95% confidence interval [CI], 0.83 to 1.03; rectal cancer: HR, 0.88; 95% CI, 0.75 to 1.04), while radiotherapy was negatively associated with cognitive impairment in rectal cancer patients (HR, 0.01; 95% CI, 0.84 to 0.99). Varying directions of the associations between regimens and cognitive impairment were detected. The adverse effect of certain chemotherapy regimens on cognition was more prominent in older adults.

**CONCLUSIONS:**

Chemotherapy and radiotherapy did not increase the risk of cognitive impairment. Older patients with low cognitive reserve could be affected by the adverse cognitive effects of chemotherapy.

## INTRODUCTION

Cancer treatment with chemotherapy and radiotherapy has continually drawn concern regarding its association with cognitive impairment. Although cognitive impairment after chemotherapy, known as “chemo-brain,” has attracted considerable attention among researchers, it remains incompletely understood [1–3]. Chemo-brain was first identified and studied in patients with breast cancer who underwent chemotherapy in the 1980s [2], and although some studies have reported potential adverse cognitive effects of chemotherapy [2], a meta-analysis of studies on breast cancer survivors in 2017 reported no overall association [1]. Research on chemo-brain in colorectal cancer, however, is relatively sparse. A single-arm study that enrolled about 80 Spanish colorectal cancer patients reported a 50% increased incidence of cognitive decline. In this study, patients received an oxaliplatin/fluorouracil regimen [4]. However, a subsequent prospective study in the United States including 362 colorectal cancer patients reported that chemotherapy did not increase the risk of cognitive impairment in cancer patients [5].

In regards to radiotherapy, fewer studies have assessed post-radiotherapy cognitive impairment—or “radio-brain”—in colorectal cancer patients. Two studies conducted in Northern Europe suggested that radiotherapy induces cognitive impairment in rectal cancer patients [6,7]. However, a Swiss study of 60 patients indicated that there was no increased risk of cognitive impairment after radiotherapy [8]. Therefore, evidence for radio-brain in rectal cancer patients is, as of yet, inconclusive, and further in-depth studies of a more extensive population are required.

It has been hypothesized that older patients with cancer are vulnerable to cognitive impairment after chemotherapy [9], as their cognitive reserve—the capacity of the brain to sustain external and internal neuropathological burdens [10]—is diminished [11]. A meta-regression from our previous systematic review and meta-analysis on cognitive decline after chemotherapy in colorectal cancer patients suggested that older colorectal cancer patients are more likely to suffer from cognitive impairment after receiving chemotherapy [12], further supporting this hypothesis. However, the studies that we previously reviewed utilized relatively small and selected populations (n<500). To support our results, epidemiological evidence from a nationwide, representative study with a large sample is needed.

In this study, we aimed to evaluate the adverse cognitive effects of cancer treatment by conducting a longitudinal analysis of a representative population of Korea. Additionally, we investigated age heterogeneity in the effects of chemotherapy and radiotherapy in patients with colorectal cancer.

## MATERIALS AND METHODS

### Participant selection

Administrative data for medical service usage among colorectal cancer patients were obtained from the Korea National Health Information Database (NHID) from January 1, 2002 to December 31, 2018. The NHID is a public database on healthcare services maintained by the National Health Insurance System (NHIS) of Korea, which is a universal health insurance system that covers the medical expenditures of approximately 98% of all Korean citizens [13]. The database includes representative and comprehensive information on medical use among Korean patients, including insurance eligibility, diagnostic codes, prescribed medications and procedures, and billing records [14].

**Figure 1:**
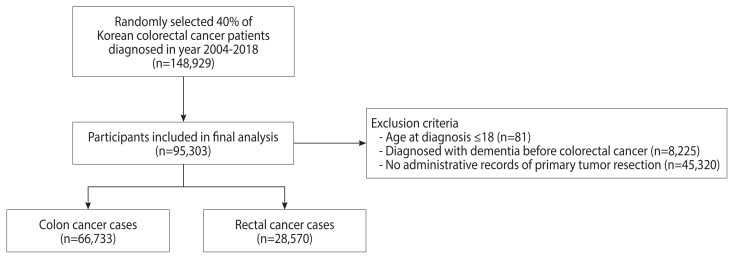
Flow chart of participant inclusion and exclusion.

Patients with 2 or more International Classification of Diseases, 10th revision (ICD-10) diagnostic codes for colorectal cancer (C18–C20) and 1 or more admission records between 2004 and 2018 were defined as colorectal cancer patients. In order to exclude prevalent cases who had been diagnosed before NHID follow-up and to include incident cases only, we set a washout period of 2 years and excluded patients diagnosed in 2002–2003 [15]. From the original database, 40% of colorectal cancer patients were randomly selected (n=148,929). We excluded patients aged 18 years or younger (n=81), those diagnosed with cognitive impairment before colorectal cancer diagnosis (n=8,225), and those without administrative records for tumor resection (n=45,320) (Figure 1). In total, 95,303 patients were included in the final analysis (66,733 colon cancer cases and 28,570 rectal cancer cases).

### Assessing colorectal cancer treatment and cognitive impairment

Cognitive impairment was defined as the presence of at least 1 ICD-10 diagnostic code for dementia or minor cognitive impairment [16,17]. The ICD-10 codes for dementia and minor cognitive impairments are listed in Supplementary Material 1. The claim codes for the cancer treatment modality, including surgical resection, chemotherapy, and radiotherapy, were reviewed and confirmed by a colorectal surgeon (CWK), a medical oncologist (HK), and 2 epidemiologists (SJJ, KK). For chemotherapy, regimens that are recommended for first-line chemotherapy in the National Comprehensive Cancer Network (NCCN) Guidelines 2019 were included for analysis (oxaliplatin, capecitabine, 5-fluorouracil [5-FU], irinotecan; the administrative codes are listed in Supplementary Material 2). Patients with at least 1 label for a chemotherapy regimen were considered to be chemotherapy recipients. The date of the first insurance claim for colorectal cancer was considered as the date of colorectal cancer onset.

### Covariates

Monthly insurance premiums were used as a proxy variable for socioeconomic status. The participants’ monthly insurance premium payment records at baseline were collected, and the participants were divided into subgroups according to quintile values of monthly insurance premiums (cut-offs: 18,700, 31,110, 44,169, and 63,749 Korean won/mo in 2004). Medical Aid recipients who did not pay a premium due to their poor economic situation were classified into a separate subgroup. The Charlson comorbidity index values at baseline were calculated to assess medical comorbidities [18]. Participants with ICD-10 diagnostic codes corresponding to individual comorbidities were considered to have those comorbidities (Supplementary Material 3).

### Statistical analysis

We classified participants according to the cancer treatment modality in accordance with claim records for cancer treatment as follows: in colon cancer, (1) primary resection only and (2) primary resection with chemotherapy; in rectal cancer, (1) primary resection only, (2) primary resection with chemotherapy, (3) primary resection and radiotherapy, and (4) primary resection with concurrent chemoradiotherapy (CCRT). Using these categories, we described the baseline characteristics of the participants by presenting mean and standard deviation values for continuous variables and numbers and percentages of participants for discrete variables.

We hypothesized that treatments were provided according to the 2019 NCCN guidelines for colorectal cancer treatment and that participants with the same treatment modality were likely to have a similar tumor burden [19,20]. To control for possible confounding by tumor burden, we excluded patients without claim codes for surgical resection, thereby excluding patients with inoperable tumors (reflecting a higher tumor burden) and chronic patients receiving palliative treatment only.

Hazard ratios (HRs) and their 95% confidence intervals (CIs) for chemotherapy and radiotherapy were estimated using a time-dependent competing risk survival analysis model, with chemotherapy and radiotherapy being considered as time-dependent variables and all-cause mortality considered as a competing risk [21–24]. To avoid immortal time bias, chemotherapy and radiotherapy were constructed as time-dependent variables, and follow-up periods were classified as primary resection with chemotherapy/radiotherapy/CCRT only after patients received the corresponding treatments [25–27]. For instance, if a patient was diagnosed with cognitive impairment after surgical resection and before chemotherapy, the follow-up period of the patient was classified as surgical resection only. Cox regression was conducted by applying the ‘proc phreg’ procedure in SAS version 9.4 (SAS Institute Inc., Cary, NC, USA). Time-dependent variables were created by ‘if else’ statements in the ‘proc phreg’ procedure. Censoring, the event (cognitive impairment), and the competing risk (all-cause mortality) were coded as 0, 1, and 2 respectively (Supplementary Material 4). We also estimated HRs for chemotherapy regimen combinations for colorectal cancer treatment, including FOLFOX (folate, 5-FU, and oxaliplatin), FOLFIRI (folate, 5-FU, and irinotecan), FOLFOXIRI (folate, 5-FU, oxaliplatin, and irinotecan), CapeOx (capecitabine and oxaliplatin), capecitabine only, 5-FU only, and irinotecan only. Considering left truncation, the date of colorectal cancer diagnosis was used as the date of follow-up initiation. Age interaction terms were added to the model to assess the moderating effects of age, and conditional HRs of chemotherapy and radiotherapy by age points were estimated [28]. All models were adjusted for age, sex, comorbidities, and the monthly insurance premium.

For sensitivity analyses, we redefined cognitive impairment cases as patients with 2 or more corresponding diagnostic codes for cognitive impairment and repeated the survival analyses. Additionally, we performed landmark analyses by conducting time-fixed Cox regression with lag times of 6 months, 12 months, and 18 months [29]. All statistical analyses were conducted using SAS version 9.4.

### Ethics statement

The study protocols were approved by the Institutional Review Board of Yonsei University Health System, Seoul, Korea (approval No. 4-2019-0425). Informed consent was waived for this study, since personal information that can be used to identify individuals registered to NHID was removed. All procedures contributing to this work complied with the ethical standards of the relevant national and institutional committees on human experimentation and with the 1975 Declaration of Helsinki, which was revised in 2008.

## RESULTS

### Characteristics of the study population

Among the 66,733 patients with colon cancer at baseline (2004), 14,146 (21.2%) received adjuvant or neoadjuvant chemotherapy until 2018. The mean follow-up duration was longer in patients who did not receive chemotherapy than in those who received it (5.57 vs. 3.21 years, p<0.001). Capecitabine, oxaliplatin, 5-FU, and irinotecan were administered to 3,228 (22.8%), 9,928 (70.2%), 7,709 (54.5%), and 2,444 (17.3%) chemotherapy recipients, respectively. The incidence rates of cognitive impairment were 22.17 per 1,000 person-years in chemotherapy non-recipients and 14.48 per 1,000 person-years in chemotherapy recipients. The all-cause mortality rates were 49.05 per 1,000 person-years in chemotherapy non-recipients and 96.52 per 1,000 person-years in patients who received chemotherapy (Table 1).

Among the 28,570 patients with rectal cancer included in the analyses, 2,604 (9.1%) received chemotherapy, 8,098 (28.3%) received radiotherapy, and 3,161 (11.1%) received CCRT before or after surgical resection. The mean follow-up duration (in years) was relatively longer in the primary resection and resection-radiotherapy combination groups and shorter in the resection-chemotherapy combination and resection-CCRT combination groups. Among 5,765 chemotherapy and CCRT recipients, capecitabine, oxaliplatin, 5-FU, and irinotecan were administered to 1,734 (30.1%), 3,289 (57.0%), 3,499 (60.7%), and 1,228 (21.3%), respectively. The incidence rate of cognitive impairment was highest in the resection only group (23.16 per 1,000 person-years), while mortality was highest in the resection-CCRT combination group (123.66 per 1,000 person-years) (Table 1).

### Effects of chemotherapy and radiotherapy on cognitive impairment

**Figure 2:**
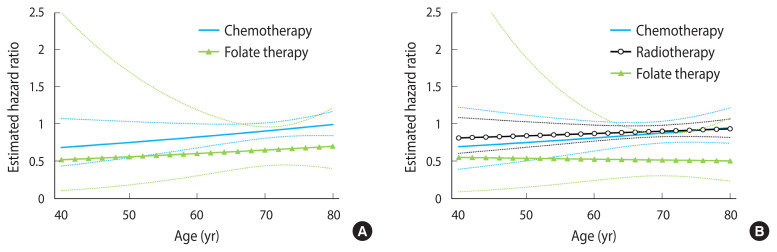
Age-specific hazard ratios of chemotherapy and radiotherapy for cognitive impairment (A) colon cancer and (B) rectal cancer.

In colon cancer patients, chemotherapy did not increase the risk of cognitive impairment (HR, 0.92; 95% CI, 0.83 to 1.03). In rectal cancer patients, neither chemotherapy (HR, 0.88; 95% CI, 0.75 to 1.04) nor radiotherapy (HR, 0.91; 95% CI, 0.84 to 0.99) was positively associated with cognitive impairment. Folate administration during chemotherapy was negatively associated with cognitive impairment in both colon cancer (HR, 0.66; 95% CI, 0.45 to 0.97) and rectal cancer (HR, 0.52; 95% CI, 0.31 to 0.88) (Table 2). The age-specific HRs of chemotherapy and radiotherapy were larger in older patients, but were not significant in any age spectra. The protective effect of folate administration was more prominent in older adult patients (Figure 2).

**Figure 3:**
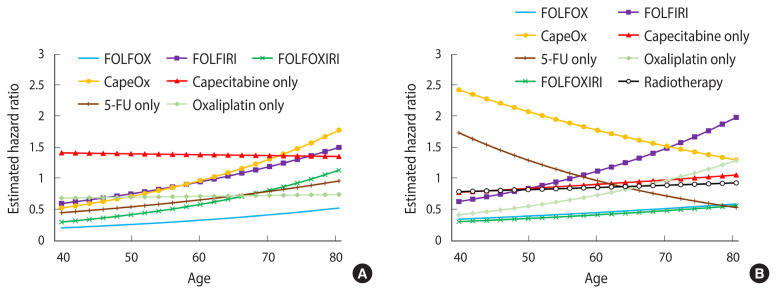
Age-specific hazard ratios of chemotherapy regimen and radiotherapy for cognitive impairment (A) colon cancer and (B) rectal cancer. FOLFOX, folate, 5-fluorouracil (5-FU), oxaliplatin; FOLFIRI, folate, 5-FU, irinotecan; FOLFOXIRI, folate, 5-FU, oxaliplatin, irinotecan; CapeOx: capecit abine, oxaliplatin.

When analyzed by regimen, the FOLFOX regimen was negatively associated with cognitive impairment in both colon cancer (HR, 0.44; 95% CI, 0.32 to 0.60) and rectal cancer (HR, 0.53; 95% CI, 0.34 to 0.82). The FOLFOXIRI regimen showed lower HRs in rectal cancer (HR, 0.49; 95% CI, 0.26 to 0.91), but not in colon cancer (HR, 0.85; 95% CI, 0.58 to 1.27). In general, patients who received the CapeOx or capecitabine-only regimen showed increased hazards for cognitive impairment, although the magnitude of the association varied by primary cancer site (Table 2). The age-specific HRs for FOLFIRI and FOLFOXIRI were higher in older patients than in younger patients, although the HRs were non-significant in all age spectra. The direction of the interaction between the effects of the CapeOx regimens and age was positive in colon cancer, but negative in rectal cancer. The capecitabine-only regimen and radiotherapy did not show significant interactions with age (Figure 3).

The sensitivity analysis showed similar results to those of the main analysis. When cognitive impairment was redefined as having 2 or more corresponding ICD-10 codes, the estimated HRs of chemotherapy and radiotherapy did not show significant differences from the main analyses (Supplementary Material 5). In landmark analyses, as the time lag increased, the estimated HRs of chemotherapy decreased and protective effects of folate therapy became more prominent (Supplemental Material 6). The interaction trends detected in landmark analyses were similar to those of the main analyses (Supplemental Material 7). Landmark analyses by regimen showed similar trends to those of the main analyses (Supplemental Material 8).

## DISCUSSION

Overall, chemotherapy and radiotherapy did not increase the risk of cognitive impairment in colorectal cancer. While the CapeOx and capecitabine-only regimens increased the risk, the FOLFOX and oxaliplatin-only regimens were negatively associated with cognitive impairment. The FOLFORI regimen was likely to be beneficial in younger patients, but increased the risk of cognitive impairment in older patients. Radiotherapy was not associated with an increased risk of cognitive impairment.

The findings of our analyses imply that the characteristics of the primary tumor, both biological and psychosocial, play an important role in the manifestation of chemotherapy-related cognitive impairment. It is well known that a significant proportion of breast cancer patients suffer from depression and anxiety, which lead to dysfunctional cognition and general fatigue [30]. A recent meta-analysis reported that around 32% of breast cancer patients suffer from depression [31]. In contrast, the results from a systematic review on depression and anxiety in colorectal cancer patients reported that only around 6% of colorectal cancer patients are affected by depression [32]. These differences in the psychological consequences of tumors, alongside variance in the biological action of chemotherapeutic agents [33,34], could have resulted in the varying directions of associations in our study. A large-scale randomized clinical trial on colorectal cancer patients and further research on the mechanisms of chemotherapy-related cognitive impairment are warranted to achieve a better understanding of these phenomena.

Adverse effects of chemotherapy appear to be more likely in older patients. Cognitive reserve, which reflects the capacity of the brain to withstand the effects of external events, toxins, or diseases that can affect cognitive function [10], is known to be associated with the vulnerability of the brain to the neurotoxicity of chemotherapeutic agents [35]. It has been postulated that cancer treatments interact with aging of the brain and accelerate cognitive decline, as brain images of cancer treatment receivers showed structural changes in the brain that were indicative of aging [36,37]. However, since several chemotherapeutic agents did not increase the risk of cognitive impairment even in older patients, care must be taken when interpreting our results.

Our study provides evidence of adverse cognitive effects of cancer treatment in colorectal patients from real-world data. Our results from a representative population of Korea suggest heterogeneity according to age in cognitive decline among colorectal cancer patients after treatment. This is one of only a few studies to utilize nationwide data in an attempt to investigate chemotherapy-related and radiotherapy-related cognitive dysfunction in colorectal cancer patients. However, this study does have some limitations. First, several chemotherapeutic agents are not covered by the NHIS, which might have caused selection bias; in particular, most non-covered regimens are for second-line treatment or palliative treatment in advanced cancer. To minimize selection bias, we excluded patients without primary cancer resection records, which could include substantial missing values.

Second, due to administrative challenges in obtaining medical records, the validity of the date of cognitive impairment onset might be questioned. As the data were collected for medical insurance administration and not for research, information on disease and mortality might have been misclassified [14]. Additionally, there might be concerns about the adequacy of assessment and treatment for cognitive impairment. During the process of cancer treatment, it is likely that patients and physicians are more focused on controlling neoplasms than on controlling complications. However, since chemo-brain is a well-known complication that is widely acknowledged by physicians and cancer survivors [3,38], it is unlikely that cognitive impairment after cancer treatment would be left uncontrolled. Additionally, the reliability of cognitive impairment diagnosis in the Korean NHID has been validated [39].

Lastly, due to the study design, selective survival bias is possible. Since the mean follow-up time was shorter in chemotherapy or radiotherapy recipients, their chances of developing cognitive impairment would be decreased. To address potential selective survival bias, we applied time-dependent competing risk survival models [21,22,24,40]. Evidence from a randomized clinical trial comparing patients with different clinical cancer stages would provide a better understanding of the true associations.

In conclusion, our results from a representative nationwide database of Korea suggest that chemotherapy and radiotherapy do not impose marked adverse cognitive effects in colorectal cancer patients. Our study provides evidence that contributes to a better understanding of the nature of cancer treatment-related cognitive impairment in colorectal cancer patients. A large-scale randomized clinical trial with a longer follow-up period is needed to thoroughly investigate the complex mechanisms of adverse effects in cancer treatment. Regular follow-up assessing cognitive function after cancer treatment could help prevent cognitive impairments in older patients with low cognitive reserve.

## Figures and Tables

**Table 1:** Characteristics of colon cancer patients from the Korea National Health Insurance Database, 2002–2018 (N=95,303)

Characteristics	Colon cancer (n=66,733)		Rectal cancer (n=28,570)			
	
	Primary resection only (n=52,587)	Resection with chemotherapy (n=14,146)	Primary resection only (n=14,707)	Resection with chemotherapy (n=2,604)	Resection with radiotherapy (n=8,098)	Resection with concurrent chemoradiotherapy (n=3,161)
Age	64.52±11.99	61.31±11.05	65.10±11.55	61.52±10.78	60.56±11.16	59.34±10.99

Male	30,588 (58.2)	8,474 (59.9)	8,940 (60.8)	1,726 (66.3)	5,282 (65.2)	2,185 (69.12)

Charlson comorbidity index						
2	1,243 (2.4)	552 (3.9)	323 (2.2)	92 (3.5)	335 (4.1)	161 (5.1)
3	3,235 (6.2)	1,376 (29.8)	830 (5.6)	273 (10.5)	818 (10.1)	424 (13.4)
4	5,218 (9.9)	2,069 (28.4)	1,419 (9.7)	407 (15.6)	1,205 (14.9)	532 (16.8)
5	7,094 (13.5)	2,440 (17.3)	1,928 (13.1)	467 (18.0)	1,359 (16.8)	566 (17.9)
6	7,999 (15.2)	2,255 (15.9)	2,295 (15.6)	428 (16.4)	1,317 (16.3)	509 (16.1)
7	7,886 (15.0)	1,940 (13.7)	2,377 (16.2)	358 (13.8)	1,090 (13.5)	377 (11.9)
8	6,848 (13.0)	1,456 (17.5)	1,952 (13.3)	246 (9.5)	790 (9.8)	259 (8.2)
9	5,222 (9.9)	997 (7.1)	1,446 (9.8)	151 (5.8)	531 (6.6)	176 (5.6)
≥10	7,842 (14.9)	1,061 (7.5)	2137 (14.5)	182 (7.0)	653 (8.1)	157 (5.0)

Insurance premium, percentile (KRW/mo)						
0	2,361 (4.5)	545 (3.8)	744 (5.1)	115 (4.4)	290 (3.6)	115 (3.6)
<20th (<18,700)	9,910 (18.8)	2,832 (20.0)	2,876 (19.6)	547 (21.0)	1,531 (18.9)	680 (21.5)
20th–40th (18,700–31,109)	9,314 (17.7)	2,682 (19.0)	2,669 (18.1)	520 (20.0)	1,561 (19.3)	655 (20.7)
40–60th (31,110–44,169)	10,280 (19.5)	2,743 (19.4)	2,882 (19.6)	516 (19.8)	1,656 (20.4)	596 (18.8)
60th–80th (44,170–63,749)	10,671 (20.3)	2,841 (20.1)	2,969 (20.2)	482 (18.5)	1,657 (20.5)	604 (19.1)
≥80th (≥63,750)	10,051 (19.1)	2,503 (17.7)	2,567 (17.4)	424 (16.3)	1,403 (17.3)	511 (16.2)

Observed person-years	5.57±3.95	3.21±2.79	6.08±4.14	3.07±2.81	6.36±3.82	3.52±2.59

Incidence rate of cognitive impairment, per 1,000 person-years	22.17	14.48	23.16	12.65	13.12	10.69

All-cause mortality rate, per 1,000 person-years	49.05	96.52	47.5	107.33	54.75	123.66

Chemotherapy regimen combination						
FOLFOX	-	4,508 (31.9)	-	776 (29.8)	-	659 (20.8)
FOLFIRI	-	891 (6.3)	-	205 (7.9)	-	212 (6.7)
FOLFOXIRI	-	1,287 (9.1)	-	284 (10.9)	-	334 (10.6)
CapeOx	-	563 (4.0)	-	114 (4.4)	-	105 (3.3)
Capecitabine only	-	2,343 (16.6)	-	436 (16.7)	-	749 (23.7)
5-fluorouracil only	-	718 (5.1)	-	223 (8.6)	-	480 (15.2)
Oxaliplatin only	-	3,311 (23.4)	-	462 (17.7)	-	317 (10.0)
Others	-	1,088 (7.7)	-	104 (4.0)	-	305 (9.6)

Radiotherapy						
Single port	-	-	-	-	4,775 (59.0)	1,023 (32.4)
Parallel port	-	-	-	-	4,970 (61.4)	1,013 (32.0)
Rotation radiation	-	-	-	-	29 (0.4)	2 (0.1)
3D radiotherapy	-	-	-	-	3,860 (47.7)	1,985 (62.9)
Brachytherapy	-	-	-	-	33 (0.4)	8 (0.2)
Density-modulated radiotherapy	-	-	-	-	320 (3.9)	685 (21.7)
Proton therapy	-	-	-	-	11 (0.1)	16 (0.5)

Values are presented as mean±standard deviation or number (%).

FOLFOX, folate, 5-fluorouracil and oxaliplatin; FOLFIRI, folate, 5-fluorouracil and irinotecan; FOLFOXIRI, folate, 5-fluorouracil, oxaliplatin and irinotecan; CapeOx, capecitabine and oxaliplatin; KRW, Korean won.

**Table 2:** Estimated hazard ratios of chemotherapy regimens and radiotherapy on cognitive impairment (n=95,303)^1^

Variables	Colon cancer (n=66,733)	Rectal cancer (n=28,570)
Surgical resection only	1.00 (reference)	1.00 (reference)

Chemotherapy, overall	0.92 (0.83, 1.03)	0.88 (0.75, 1.04)

Folate therapy, overall	0.66 (0.45, 0.97)	0.52 (0.31, 0.88)

Chemotherapy, by regimen		
FOLFOX	0.44 (0.32, 0.60)	0.53 (0.34, 0.82)
FOLFIRI	1.22 (0.77, 1.94)	1.43 (0.87, 2.35)
FOLFOXIRI	0.85 (0.58, 1.27)	0.49 (0.26, 0.91)
CapeOx	1.33 (0.82, 2.18)	1.60 (0.93, 2.76)
Capecitabine only	1.37 (1.16, 1.62)	1.01 (0.76, 1.34)
5-FU only	0.86 (0.54, 1.37)	0.71 (0.47, 1.05)
Oxaliplatin only	0.73 (0.60, 0.89)	0.91 (0.65, 1.29)

Values are presented as hazard ratio (95% confidence interval).

Overall, chemotherapy and radiotherapy were not significantly associated with cognitive impairment in colorectal cancer. Folate therapy was negatively associated with cognitive impairment. When analyzed by regimen, FOLFOX and FOLFOXIRI decreased the risk of cognitive impairment, while the CapeOx and capecitabine-only regimens were positively associated with cognitive impairment.

FOLFOX, folate, 5-fluorouracil (5-FU). oxaliplatin; FOLFIRI, folate, 5-FU, irinotecan; FOLFOXIRI, folate, 5-FU, oxaliplatin, irinotecan; CapeOx: capecitabine, oxaliplatin.

1 All models are adjusted for age, sex, Charlson comorbidity index, and monthly insurance premium.
