# Supplementary material for: Effect of chemotherapy and radiotherapy on cognitive impairment in colorectal cancer: evidence from Korean National Health Insurance Database Cohort
Source: Epidemiol Health. 2021 Nov 2;43:e2021093. doi: 10.4178/epih.e2021093 (PMC8920736; doi:10.4178/epih.e2021093)
Supplement: Supplementary file 1 [file epih-43-e2021093-suppl1.docx]

**Supplementary Material 1.** **Definition of colorectal cancer and cognitive disorders by ICD-10 diagnostic codes.**

| **ICD-10 codes** | **Diseases** |
| --- | --- |
| **Colorectal cancer** | |
| C18 | Malignant neoplasm of colon |
| C19 | Malignant neoplasm of rectosigmoid junction |
| C20 | Malignant neoplasm of rectum |
| **Cognitive disorders** |  |
| F00 | Dementia in Alzheimer disease |
| F01 | Vascular dementia |
| F02 | Dementia in other diseases classified elsewhere |
| F03 | Unspecified dementia |
| F06.7 | Mild cognitive impairment |
| G30 | Alzheimer disease |
| G31.00 | Behavioral variant frontotemporal dementia |
| G31.82 | Dementia with Lewy bodies |
