# Supplementary material for: Effect of chemotherapy and radiotherapy on cognitive impairment in colorectal cancer: evidence from Korean National Health Insurance Database Cohort
Source: Epidemiol Health. 2021 Nov 2;43:e2021093. doi: 10.4178/epih.e2021093 (PMC8920736; doi:10.4178/epih.e2021093)
Supplement: Supplementary file 2 [file epih-43-e2021093-suppl2.docx]

**Supplementary Material 2. Insurance fee codes for colorectal cancer treatments.**

| **Insurance claim fee codes** | **Medications or procedures** |
| --- | --- |
| **Primary resection: colon** | |
| QA671 | Right or left hemicolectomy, without lymph node dissection. |
| QA672 | Right or left hemicolectomy, with lymph node dissection. |
| Q1261 | Subtotal colectomy, without lymph node dissection. |
| Q1262 | Subtotal colectomy, with lymph node dissection. |
| QA671 | Total colectomy, without lymph node dissection. |
| QA672 | Subtotal colectomy, with lymph node dissection. |
| QA673 | Segmental colectomy, without lymph node dissection. |
| Q2673 | Segmental colectomy, with lymph node dissection. |
| QA679 | Colectomy with proximal colostomy and distal stump,  without lymph node dissection. |
| Q2679 | Colectomy with proximal colostomy and distal stump,  with lymph node dissection. |
| **Primary resection: rectum** |  |
| QA921 | Anterior resection, without lymph node dissection. |
| Q2921 | Anterior resection, with lymph node dissection. |
| QA922 | Low anterior resection, without lymph node dissection. |
| Q2922 | Low anterior resection, with lymph node dissection. |
| Q2927 | Low anterior resection, with colonic J pouch formation. |
| QA923 | Abdominoperineal/abdominosacral resection, without lymph node dissection. |
| Q2923 | Abdominoperineal/abdominosacral resection, with lymph node dissection. |
| **Chemotherapy** |  |
| 122701ATB | Capecitabine, 0.15g |
| 122702ATB | Capecitabine, 0.5g |
| 205830BIJ | Oxaliplatin, 0.05g |
| 205832BIJ | Oxaliplatin, 0.2g |
| 205834BIJ | Oxaliplatin, 0.1g |
| 205803BIJ | Oxaliplatin, 0.15g |
| 161430BIJ | 5-fluorouracil (5-FU), 0.25g |
| 161431BIJ | 5-FU, 0.5g |
| 161432BIJ | 5-FU, 1g |
| 177430BIJ | Irinotecan, 0.04g |
| 177431BIJ | Irinotecan, 0.1g |
| 177433BIJ | Irinotecan, 0.2g |
| 177435BIJ | Irinotecan, 0.3g |
| **Radiotherapy** |  |
| HD051 | Low energy teletherapy, single port. |
| HD052 | Middle energy teletherapy, single port. |
| HD053 | High energy teletherapy, single port. |
| HD054 | Low energy teletherapy, parallel opposed ports. |
| HD055 | Middle energy teletherapy, parallel opposed ports. |
| HD056 | High energy teletherapy, parallel opposed ports. |
| HD057 | Low energy rotational irradiation |
| HD058 | Middle energy rotational irradiation |
| HD059 | High energy rotational irradiation |
| HD061 | 3-Dimensional conformal therapy |
| HD080 | Brachytherapy |
| HD081 – HD082 | Intracavity therapy, high dose rate |
| HD083 – HD084 | Intracavity therapy, low dose rate |
| HD085 – HD086 | Interstitial/intraluminal therapy, high dose rate |
| HD087 – HD088 | Interstitial/intraluminal therapy, high dose rate |
| HD121 | Proton therapy |
| HZ271 | Intensity modulated radiation therapy |
| **Folate therapy** |  |
| 566132BIJ | Leucovorin, 0.2g |
| 566134BIJ | Leucovorin, 0.35g |
| 622630BIJ | Levoleucovorin, 0.05g |
| 622631BIJ | Levoleucovorin, 0.2g |
| 622632BIJ | Levoleucovorin, 0.45g |
