# Supplementary material for: Effect of chemotherapy and radiotherapy on cognitive impairment in colorectal cancer: evidence from Korean National Health Insurance Database Cohort
Source: Epidemiol Health. 2021 Nov 2;43:e2021093. doi: 10.4178/epih.e2021093 (PMC8920736; doi:10.4178/epih.e2021093)
Supplement: Supplementary file 3 [file epih-43-e2021093-suppl3.docx]

**Supplementary Material 3. Definition of comorbidities by ICD-10 diagnostic codes.**

| **ICD-10 codes** | **Diseases** |
| --- | --- |
| **Myocardial infarction** | |
| I21 | Acute myocardial infarction |
| I22 | Subsequent myocardial infarction |
| I25.2 | Old myocardial infarction |
| **Heart failure** |  |
| I50 | Heart failure |
| I11.0 | Hypertensive heart disease with (congestive) heart failure |
| I13.2 | Hypertensive heart and renal disease with both (congestive) heart failure and renal failure |
| I42.0 | Congestive cardiomyopathy |
| **Peripheral vascular disease** |  |
| I70 | Atherosclerosis |
| I71 | Aortic aneurysm and dissection |
| I73.1 | Thromboangiitis obliterans [Buerger] |
| I73.8 | Other specified peripheral vascular diseases |
| I73.9 | Peripheral vascular disease, unspecified |
| I77.1 | Stricture of artery |
| I79 | Disorders of arteries, arterioles and capillaries in diseases classified elsewhere |
| K55 | Vascular disorders of intestine |
| **Cerebrovascular disease** |  |
| G45 | Transient cerebral ischaemic attacks and related syndromes |
| G46 | Vascular syndromes of brain in cerebrovascular diseases |
| I60 | Subarachnoid haemorrhage |
| I61 | Intracerebral haemorrhage |
| I62 | Other nontraumatic intracranial haemorrhage |
| I63 | Cerebral infarction |
| I64 | Stroke, not specified as haemorrhage or infarction |
| I65 | Occlusion and stenosis of precerebral arteries, not resulting in cerebral infarction |
| I66 | Occlusion and stenosis of cerebral arteries, not resulting in cerebral infarction |
| I67 | Other cerebrovascular diseases |
| I68 | Cerebrovascular disorders in diseases classified elsewhere |
| I69 | Sequelae of cerebrovascular disease |
| **Dementia** |  |
| F00 | Dementia in Alzheimer’s disease |
| F01 | Vascular dementia |
| F02 | Dementia in other diseases classified elsewhere |
| F03 | Unspecified dementia |
| F05.1 | Delirium superimposed on dementia |
| G30 | Alzheimer’s disease |
| G31.00 | Behavioral variant frontotemporal dementia |
| G31.92 | Dementia with Lewy bodies |
| **Chronic obstructive pulmonary disease** | |
| J42 | Unspecified chronic bronchitis |
| J43 | Emphysema |
| J44 | Other chronic obstructive pulmonary disease |
| I27 | Other pulmonary heart diseases |
| J68.4 | Chronic respiratory conditions due to chemicals, gases, fumes and vapours |
| J70.1 | Chronic and other pulmonary manifestations due to radiation |
| J70.3 | Chronic drug-induced interstitial lung disorders |
| **Connective tissue disease** |  |
| M05 | Seropositive rheumatoid arthritis |
| M06 | Other rheumatoid arthritis |
| M30 | Polyarteritis nodosa and related conditions |
| M31 | Other necrotizing vasculopathies |
| M32 | Systemic lupus erythematosus |
| M33 | Dermatopolymyositis |
| M34 | Multiple sclerosis |
|  |  |
| M35 | Other systemic involvement of connective tissue |
| M36 | Systemic disorders of connective tissue in diseases classified elsewhere |
| **Mild iver disease: should not have ICD-10 diagnostic codes indicating ‘moderate to severe liver disease’.** | |
| B18 | Chronic viral hepatitis |
| K70 | Alcoholic liver disease |
| K71 | Toxic liver disease |
| K72 | Hepatic failure, NEC |
| K73 | Chronic hepatitis, NEC |
| K74 | Fibrosis and cirrhosis of liver |
| K76 | Other diseases of liver |
| **Moderate to severe liver disease** | |
| K70.4 | Alcoholic hepatic failure |
| K71.1 | Toxic liver diseas with hepatic necrosis |
| K76.6 | Portal hypertension |
| K76.7 | Hepatorenal syndrome |
| I85 | Oesophageal varices |
| I86.4 | Gastric varices |
| I98.2 | Oesophageal varices without bleeding in diseases classified elsewhere |
| **Diabetes mellitus without complication: should not have ICD-10 diagnostic codes indicating ‘diabetes mellitus with complication’.** | |
| E10 | Insulin-dependent diabetes mellitus |
| E11 | Non-insulin dependent diabetes mellitus |
| E12 | Malnutrition-related diabetes mellitus |
| E13 | Other specified diabetes mellitus |
| E14 | Unspecified diabetes mellitus |
| **Diabetes mellitus with complication** |  |
| E10.0 | Insulin-dependent diabetes mellitus with coma |
| E10.1 | Insulin-dependent diabetes mellitus with acidosis |
| E10.2 | Insulin-dependent diabetes mellitus with renal complications |
| E10.3 | Insulin-dependent diabetes mellitus with retinopathy |
| E10.4 | Insulin-dependent diabetes mellitus with neurologic complications |
| E10.5 | Insulin-dependent diabetes mellitus with peripheral circulatory complications |
| E10.6 | Insulin-dependent diabetes mellitus with other specified complications |
| E11.0 | Non-insulin-dependent diabetes mellitus with coma |
| E11.1 | Non-insulin-dependent diabetes mellitus with acidosis |
| E11.2 | Non-insulin-dependent diabetes mellitus with renal complications |
| E11.3 | Non-insulin-dependent diabetes mellitus with retinopathy |
| E11.4 | Non-insulin-dependent diabetes mellitus with neurologic complications |
| E11.5 | Non-insulin-dependent diabetes mellitus with peripheral circulatory complications |
| E11.6 | Non-insulin-dependent diabetes mellitus with other specified complications |
| E12.0 | Malnutrition-related diabetes mellitus with coma |
| E12.1 | Malnutrition-related diabetes mellitus with acidosis |
| E12.2 | Malnutrition-related diabetes mellitus with renal complications |
| E12.3 | Malnutrition-related diabetes mellitus with retinopathy |
| E12.4 | Malnutrition-related diabetes mellitus with neurologic complications |
| E12.5 | Malnutrition-related diabetes mellitus with peripheral circulatory complications |
| E12.6 | Malnutrition-related diabetes mellitus with other specified complications |
| E13.0 | Other specified diabetes mellitus with coma |
| E13.1 | Other specified diabetes mellitus with acidosis |
| E13.2 | Other specified diabetes mellitus with renal complications |
| E13.3 | Other specified diabetes mellitus with retinopathy |
| E13.4 | Other specified diabetes mellitus with neurologic complications |
| E13.5 | Other specified diabetes mellitus with peripheral circulatory complications |
| E13.6 | Other specified diabetes mellitus with other specified complications |
| E14.0 | Unspecified diabetes mellitus with coma |
| E14.1 | Unspecified diabetes mellitus with acidosis |
| E14.2 | Unspecified diabetes mellitus with renal complications |
| E14.3 | Unspecified diabetes mellitus with retinopathy |
| E14.4 | Unspecified diabetes mellitus with neurologic complications |
| E14.5 | Unspecified diabetes mellitus with peripheral circulatory complications |
| E14.6 | Unspecified diabetes mellitus with other specified complications |
| **Moderate to severe chronic kidney disease** | |
| I12.0 | Hypertensive renal disease with renal failure |
| I13 | Hypertensive heart and renal disease |
| N18 | Chronic kidney disease |
| N19 | Unspecified kidney failure |
| **Neoplasms (both solid and hematologic): All ‘C’ codes** | |
| **Metastatic neoplasms** |  |
| C77 | Secondary and unspecified malignant neoplasm of lymph nodes |
| C78 | Secondary malignant neoplasm of respiratory and digestive organs |
| C79 | Secondary malignant neoplasm of other and unspecified sites |
| C80 | Malignant neoplasm without specification of site |
| **Human immunodeficiency virus (HIV) infection and acquired immunodeficiency syndrome (AIDS)** | |
| B20 | Human immunodeficiency virus [HIV] disease resulting in infectious and parasitic diseases |
| B21 | Human immunodeficiency virus [HIV] disease resulting in malignant neoplasms |
| B22 | Human immunodeficiency virus [HIV] disease resulting in other specified diseases |
| B23 | Human immunodeficiency virus [HIV] disease resulting in other conditions |
| B24 | Unspecified human immunodeficiency virus [HIV] disease |
