# Supplementary material for: Effect of chemotherapy and radiotherapy on cognitive impairment in colorectal cancer: evidence from Korean National Health Insurance Database Cohort
Source: Epidemiol Health. 2021 Nov 2;43:e2021093. doi: 10.4178/epih.e2021093 (PMC8920736; doi:10.4178/epih.e2021093)
Supplement: Supplementary file 4 [file epih-43-e2021093-suppl4.docx]

**Supplementary Material 4. SAS code for time-dependent competing risk Cox regression.**

/*Colon cancer*/

proc phreg data=cb.colonnewfinal;

class incomegp(ref=’5’) sex_type(ref=’1’) cci(ref=’2’) / param=’ref’;

model (startdate, finaloutdate)*dementiafinal(0,2) = age sex_type cci incomegp x y;

if (ctx=1 and chemodate<=finaloutdate) then x=1; else x=0;

if (folate=1 and folatedate<=finaloutdate) then y=1; else y=0;

hazardratio ‘chemotherapy’ x/cl=wald;

hazardratio ‘folate therapy’ y/cl=wald;

run;

/*Rectal cancer*/

proc phreg data=cb.rectalnewfinal;

class incomegp(ref=’5’) sex_type(ref=’1’) cci(ref=’2’) / param=’ref’;

model (startdate, finaloutdate)*dementiafinal(0,2) = age sex_type cci incomegp x y z;

if (ctx=1 and chemodate<=finaloutdate) then x=1; else x=0;

if (folate=1 and folatedate<=finaloutdate) then y=1; else y=0;

if (rt=1 and radiodate<=finaloutdate) the z=1; else z=0;

hazardratio ‘chemotherapy’ x/cl=wald;

hazardratio ‘folate therapy’ y/cl=wald;

run;
