# Supplementary material for: Effect of chemotherapy and radiotherapy on cognitive impairment in colorectal cancer: evidence from Korean National Health Insurance Database Cohort
Source: Epidemiol Health. 2021 Nov 2;43:e2021093. doi: 10.4178/epih.e2021093 (PMC8920736; doi:10.4178/epih.e2021093)
Supplement: Supplementary file 5 [file epih-43-e2021093-suppl5.docx]

**Supplementary Material 5. Estimated hazard ratios of chemotherapy regimen combination and radiotherapy on cognitive impairment. Cognitive impairment cases were redefined as patients with two or more corresponding ICD-10 codes for cognitive impairment for sensitivity analyses (N = 95,303).**

|  | **Colon cancer (N=66,733)** | **Rectal cancer (N=28,570)** |
| --- | --- | --- |
| **Surgical resection only** | ref | ref |
| **Chemotherapy, overall** | 0.90 (0.79 - 1.02) | 0.84 (0.69 - 1.03) |
| **Folate therapy, overall** | **0.59 (0.38 - 0.93)** | 0.62 (0.34 - 1.13) |
|  |  |  |
| **Chemotherapy, by regimen** |  |  |
| FOLFOX | **0.36 (0.25 - 0.52)** | **0.53 (0.35 - 0.82)** |
| FOLFIRI | 1.04 (0.61 - 1.75) | 1.35 (0.83 - 2.22) |
| FOLFOXIRI | 0.81 (0.53 - 1.25) | 0.45 (0.24 - 0.84) |
| CapeOx | 1.19 (0.69 - 2.04) | 1.42 (0.83 - 2.45) |
| Capecitabine only | **1.31 (1.09 - 1.57)** | 1.06 (0.80 - 1.41) |
| 5-FU only | 0.78 (0.44 - 1.38) | 0.83 (0.56 - 1.24) |
| Oxaliplatin only | **0.60 (0.48 - 0.75)** | 0.79 (0.56 - 1.11) |

All models are adjusted for age, sex, Charlson Comorbidity Index, and monthly insurance premium. FOLFOX: folate, 5-fluorouracil (5-FU). oxaliplatin; FOLFIRI: folate, 5-FU, irinotecan; FOLFOXIRI: folate, 5-FU, oxaliplatin, irinotecan; CapeOx: capecitabine, oxaliplatin; HR, hazard ratio; CI, confidence interval.
