# Supplementary material for: Effect of chemotherapy and radiotherapy on cognitive impairment in colorectal cancer: evidence from Korean National Health Insurance Database Cohort
Source: Epidemiol Health. 2021 Nov 2;43:e2021093. doi: 10.4178/epih.e2021093 (PMC8920736; doi:10.4178/epih.e2021093)
Supplement: Supplementary file 6 [file epih-43-e2021093-suppl6.docx]

**Supplementary Material 6. Estimated hazard ratios of chemotherapy regimen combination and radiotherapy on cognitive impairment with landmarks of 6, 12 and 18 months.**

| **Time lag** | **6 months** | **12 months** | **18 months** |
| --- | --- | --- | --- |
| **Colon cancer** | | | |
| **N** | 66,173 | 65,819 | 65,525 |
| **Chemotherapy, overall** | 0.90 (0.80 - 1.02) | **0.84 (0.75 - 0.96)** | **0.77 (0.67 - 0.88)** |
| **Folate therapy, overall** | **0.60 (0.40 - 0.91)** | **0.47 (0.29 - 0.77)** | **0.38 (0.21 - 0.67)** |
|  |  |  |  |
| **Chemotherapy, by regimen** |  |  |  |
| FOLFOX | **0.39 (0.28 - 0.55)** | **0.26 (0.17 - 0.39)** | **0.44 (0.32 - 0.60)** |
| FOLFIRI | 1.15 (0.70 - 1.89) | 1.12 (0.67 - 1.86) | 1.22 (0.77 - 1.94) |
| FOLFOXIRI | 0.73 (0.47 - 1.13) | **0.61 (0.37 - 0.99)** | 0.85 (0.58 - 1.27) |
| CapeOx | 1.44 (0.88 - 2.36) | 1.34 (0.79 - 2.26) | 1.33 (0.82 - 2.18) |
| Capecitabine only | **1.36 (1.14 - 1.63)** | **1.30 (1.08 - 1.57)** | **1.37 (1.16 - 1.62)** |
| 5-FU only | 0.78 (0.47 - 1.30) | 0.65 (0.37 - 1.15) | 0.86 (0.54 - 1.37) |
| Oxaliplatin only | **0.73 (0.60 - 0.90)** | **0.73 (0.59 - 0.90)** | **0.73 (0.60 - 0.89)** |
| **Rectal cancer** | | | |
| **N** | 28,338 | 28,153 | 28,008 |
| **Chemotherapy, overall** | 0.86 (0.73 - 1.02) | **0.78 (0.65 - 0.93)** | **0.75 (0.62 - 0.91)** |
| **Folate therapy, overall** | **0.38 (0.21 - 0.70)** | **0.28 (0.13 - 0.59)** | **0.29 (0.14 - 0.63)** |
|  |  |  |  |
| **Chemotherapy, by regimen** |  |  |  |
| FOLFOX | **0.41 (0.25 - 0.68)** | **0.24 (0.12 - 0.46)** | **0.52 (0.34 - 0.81)** |
| FOLFIRI | 1.46 (0.89 - 2.40) | 1.30 (0.77 - 2.20) | 1.42 (0.87 - 2.33) |
| FOLFOXIRI | **0.45 (0.23 - 0.87)** | **0.40 (0.20 - 0.81)** | **0.48 (0.26 - 0.90)** |
| CapeOx | 1.67 (0.97 - 2.87) | 1.60 (0.90 - 2.81) | 1.58 (0.92 - 2.73) |
| Capecitabine only | 1.02 (0.76 - 1.36) | 0.95 (0.70 - 1.29) | 0.99 (0.74 - 1.31) |
| 5-FU only | **0.58 (0.38 - 0.91)** | **0.50 (0.31 - 0.81)** | 0.68 (0.46 - 1.01) |
| Oxaliplatin only | 0.90 (0.63 - 1.29) | 0.86 (0.59 - 1.25) | 0.92 (0.65 - 1.30) |
|  |  |  |  |
| **Radiotherapy** | 0.94 (0.86 - 1.02) | 0.94 (0.86 - 1.03) | 0.93 (0.85 - 1.03) |

All models are adjusted for age, sex, Charlson Comorbidity Index, and monthly insurance premium. FOLFOX: folate, 5-fluorouracil (5-FU). oxaliplatin; FOLFIRI: folate, 5-FU, irinotecan; FOLFOXIRI: folate, 5-FU, oxaliplatin, irinotecan; CapeOx: capecitabine, oxaliplatin; HR, hazard ratio; CI, confidence interval.
