# Supplementary material for: Effect of chemotherapy and radiotherapy on cognitive impairment in colorectal cancer: evidence from Korean National Health Insurance Database Cohort
Source: Epidemiol Health. 2021 Nov 2;43:e2021093. doi: 10.4178/epih.e2021093 (PMC8920736; doi:10.4178/epih.e2021093)
Supplement: Supplementary file 7 [file epih-43-e2021093-suppl7.docx]

**A) Colon cancer, time lag = 6 months B) Rectal cancer, time lag = 6 months**

**C) Colon cancer, time lag = 12 months D) Rectal cancer, time lag = 12 months**

**E) Colon cancer, time lag = 18 months F) Rectal cancer, time lag = 18 months**

**Supplementary Material 7.** Estimated hazard ratios of chemotherapy and radiotherapy on cognitive impairment under landmark analyses. Landmark analyses with time lags of 6, 12 and 18 months showed no significant differences with main analyses. Protective effect of folate therapy was more prominent in older patients.
