# Supplementary material for: Effect of chemotherapy and radiotherapy on cognitive impairment in colorectal cancer: evidence from Korean National Health Insurance Database Cohort
Source: Epidemiol Health. 2021 Nov 2;43:e2021093. doi: 10.4178/epih.e2021093 (PMC8920736; doi:10.4178/epih.e2021093)
Supplement: Supplementary file 8 [file epih-43-e2021093-suppl8.docx]

**A) Colon cancer, time lag = 6 months B) Rectal cancer, time lag = 6 months**

**C) Colon cancer, time lag = 12 months D) Rectal cancer, time lag = 12 months**

**E) Colon cancer, time lag = 18 months F) Rectal cancer, time lag = 18 months**

**Supplementary Material 8.** Estimated hazard ratios of chemotherapy regimen combination and radiotherapy on cognitive impairment under landmark analyses. Trends of effect modification by age in landmark analyses did not significantly vary from main analyses. Most regimens showed positive interaction with age, while negative interaction was detected in 5-FU only regimen and CapeOx regimen (rectal cancer only).
